# Autoimmune encephalitis: clinical spectrum and management

**DOI:** 10.1136/practneurol-2020-002567

**Published:** 2021-06-09

**Authors:** Christopher E Uy, Sophie Binks, Sarosh R Irani

**Affiliations:** 1 Oxford Autoimmune Neurology Group, Nuffield Department of Clinical Neurosciences, Oxford, UK; 2 Department of Neurology, Oxford University Hospitals NHS Foundation Trust, Oxford, UK

**Keywords:** neuroimmunology, clinical neurology, epilepsy, immunology, limbic system

## Abstract

Autoimmune encephalitis defines brain inflammation caused by a misdirected immune response against self-antigens expressed in the central nervous system. It comprises a heterogeneous group of disorders that are at least as common as infectious causes of encephalitis. The rapid and ongoing expansion of this field has been driven by the identification of several pathogenic autoantibodies that cause polysymptomatic neurological and neuropsychiatric diseases. These conditions often show highly distinctive cognitive, seizure and movement disorder phenotypes, making them clinically recognisable. Their early identification and treatment improve patient outcomes, and may aid rapid diagnosis of an underlying associated tumour. Here we summarise the well-known autoantibody-mediated encephalitis syndromes with neuronal cell-surface antigens. We focus on practical aspects of their diagnosis and treatment, offer our clinical experiences of managing such cases and highlight more basic neuroimmunological advances that will inform their future diagnosis and treatments.

## Introduction

Autoimmune encephalitis comprises a group of disorders in which the host immune system targets self-antigens expressed in the central nervous system (CNS).^1^ Some of the best-characterised diseases are associated with autoantibodies that target neuroglial antigens (table 1). These autoantibodies are considered pathogenic because they are directed against the extracellular—and hence *in vivo* exposed—domains of their target antigens.^2–4^ This fundamental property has led to much interest and excitement surrounding this rapidly expanding field, with new autoantibody targets described most years. Many established antigens are key synaptic proteins, ion channels or receptors, meaning that the extracellular domain-targeting autoantibodies are likely to directly modulate critical physiological processes.

**Table 1:** Demographic, clinical and paraclinical features of neuronal autoantibody syndromes

Neuronal auto-antibody (Ref.) and predominant IgG subclass	Median age, years (range)	Sex ratio (M:F)	Clinical features	MR brain scan findings	CSF findings	EEG findings	Other investigations	Immunotherapy response and outcomes
NMDAR^10 25 43 56^ IgG1	21 (2 months–85 years)	1:4	Encephalitis with prominent polysymptomatic neuropsychiatric presentation, polymorphic movement disorder, language disorder, autonomic dysfunction, coma and central apnoea.	70%–80% normal or non-specific, with a typical limbic encephalitis in a minority.	80% abnormal (lymphocytic pleocytosis, unpaired oligoclonal bands common).	90% abnormal (slowing most common, 20% epileptiform abnormalities, rarely extreme delta brush pattern).	Ovarian teratoma in 60% of adult, female patients. After HSV encephalitis, particularly children can develop NMDAR (and other neuronal surface) autoantibodies.	~50% improve in 4 weeks with first line immunotherapy (IT). ~70% of non-responders improve soon after 2nd line IT. Improvement up to 24 months, with 80% reaching mRS 0–2. 10%–15% relapse risk—reduced by IT and tumour removal ~5% mortality.
LGI1*^33 49 57^ IgG4	64 (31–84)	2:1	Limbic encephalitis with frequent focal seizures, including characteristic facio-brachial dystonic seizures.	~75% abnormal. ~40% increased signal/swelling in medial temporal lobes (unilateral >bilateral).	~25% abnormal (mild pleocytosis with elevated protein).	~50% abnormal (~30% epileptiform abnormal, ~20% focal slowing).	>90% with HLA-DRB1*07:01. Hyponatraemia common (~70%).	At 2 years, 1/3 fully recovered, 1/3 functionally independent but unable to work, 1/3 severely disabled or dead. Relapses in 20%–30%; associated with poor outcomes.
CASPR2*^30 49 55^ IgG4	66 (25–77)	9:1	*Main syndromes*: peripheral nerve hyperexcitability, limbic encephalitis and Morvan’s syndrome.	~30% increased signal in medial temporal lobes.	~30% abnormal (pleocytosis, elevated protein±oligoclonal bands).	~70% abnormal (40% epileptiform abnormal).	HLA-DRB1*11:01. Thymoma in ~20% (often with LGI1 antibodies in addition) Electromyography may demonstrate hyperexcitability (fasciculations, myokymia).	~50% with good or full response to tumour therapy/IT. ~45% with partial IT response. ~25% relapse.
GABA_A_R^20 21^ IgG1	40 (2 months–88 years)	1:1	Encephalitis with frequent status epilepticus.	>80% cortical and subcortical FLAIR signal abnormalities involving 2+ brain regions.	25–50% lymphocytic pleocytosis±oligoclonal bands and elevated protein.	>80% abnormal (encephalopathy with ictal abnormalities).	Thymoma ~30%.	IT-responsive, however, mortality due to status epilepticus or related complications ~10–20%.
GABA_B_R^22^ IgG1	61 (16–77)	1.5:1	Limbic encephalitis with prominent seizures.	~70% abnormal (45% increased signal in medial temporal lobes.	~80% lymphocytic pleocytosis.	~75% with ictal abnormalities.	Tumours in ~50% (mostly SCLC).	~90% show response to IT, those with tumour have poorer prognosis with recurrent neurological symptoms and higher mortality.
AMPAR^58^	Mean 53.1 (14–92)	2:1	Limbic encephalitis with prominent confusion, amnesia, seizures and psychiatric/behavioural symptoms.	~85% abnormal (67% with bilateral mesial temporal involvement).	~70% abnormal.	45% abnormal.	Tumour identified in ~70% (thymus, SCLC, breast, ovary).	Most patients showed improvement from peak of disease, median mRS=1 in survivors. ~15% of reported patients died (commonly due to complications from malignancy).
DPPX^27^	53 (13–76)	1.5:1	Multifocal encephalitis with myoclonus, tremors and hyperekplexia, prominent diarrhoea/weight loss.	100% normal or non-specific.	~30% abnormal (mild pleocytosis and elevated protein).	~70% abnormal (focal or diffuse slowing).	~10% with B-cell neoplasm (gastrointestinal follicular lymphoma,; leukaemia).	60%–70% improve with IT.
GlyR^28^ IgG1/3	50 (1–75)	1:1	*3 main syndromes*: stiff-person spectrum disorder PERM (progressive encephalopathy with rigidity and myoclonus limbic encephalitis).	*Brain*: temporal lobe inflammation in 5%, abnormal ~30%, mostly non-specific. *Cord*: ~20% (mostly short/patchy lesions, 5% longitudinally extensive lesion).	~40% pleocytosis, 20% oligoclonal bands.	~70% abnormal (55% diffuse slowing, 15% focal epileptic abnormal, 5% focal slowing).	EMG abnormal 60% (continuous motor unit activity, spontaneous or stimulus-induced activity, neuromyotonia) Thymoma in 15%.	~10% mortality in initial case series. Good outcomes in survivors with median mRS=1 at latest follow-up. Duration of follow-up 18 months–7 years, 82% treated with IT.
MOG^17–19 59^	37 (1–74)	1:1	Optic neuritis, transverse myelitis, brainstem encephalitis, encephalitis.	*Brain*: ~75% abnormal (bilateral poorly demarcated subcortical lesions), ~30% brainstem involvement. *Cord*: ~50% abnormal, mixed STM/LTM with frequent conus medullaris involvement. *Orbit*: extensive, often bilateral, optic nerve lesions with frequent chiasmal involvement.	~60% lymphocytic pleocytosis oligoclonal bands uncommon.	Not reported.	Visual evoked potentials may show evidence of previous optic neuritis.	~75% have good response to corticosteroids. ~60% make a full or good recovery. Relapses are common.
IgLON5^32 60^ IgG1/4	64 (46–83)	1:1	*4 main syndromes*: sleep disorder (REM and NREM parasomnias, sleep apnoea) Bulbar syndrome Progressive supranuclear palsy-like syndrome Cognitive syndrome±chorea.	~80% normal/ non-specific. ~15% brainstem atrophy. 5% bilateral hippocampal atrophy.	30% CSF pleocytosis. 50% elevated protein (mean 64 mg/dL, 52–192). ~10% unpaired oligoclonal bands.	Not reported.	HLA-DRB1*10:01/HLA-DQB*05:01 alleles in 87%. No history of autoimmunity or cancer in 91%.	Up to 50% respond to initial IT but far fewer have a sustained response. Response better with combination therapy vs monotherapy (67% vs 32%) and better for second-line vs first-line therapy (59% vs 32%).
Neurexin-3α^61^	44 (23–57)	1:2	Encephalitis.	20% mesial temporal T2/FLAIR signal abnormal.	100% abnormal (pleocytosis, elevated Ig index).	Not reported		40% mortality despite IT, remaining 60% partial recovery in initial series.

*LGI1-antibodies and CASPR2-antibodies were historically classified as antibodies against the Voltage-Gated Potassium Channel.

AMPAR, α-amino-3-hydroxy-5-methyl-4-isoxazolepropionic acid receptor; CASPR2, contact-associated protein 2; CSF, cerebrospinal fluid; DPPX, dipeptidyl peptidase-like protein 6; EEG, electroencephalogram; GABA_A/B_R, gamma aminobutyric acid; GlyR, glycine receptor; HSV, herpes simplex virus; IgLON5, immunoglobulin-like cell-adhesion molecule 5; IT, immunotherapy; L, long-segment; LGI1, leucine-rich glioma inactivated protein 1; MOG, myelin-oligodendrocyte glycoprotein; mRS, modified Rankin score; NMDAR, N-methyl-D-aspartate receptor; (N)REM, (non)-rapid eye-movement sleep; S, short-segment; SCLC, Small Cell Lung Cancer; TM, transverse myelitis.

This field is of major clinical importance to all neurologists because these patients present with a wide variety of neurological features and typically respond to immunotherapies. Therefore, these conditions are often considered ‘not to miss’ diagnoses, with defined pathogenic agents that can present to cognitive, movement disorder, epilepsy, psychiatry and peripheral nerve clinics.

In this pragmatic review, which reflects our experience of managing >200 cases with surface-directed autoantibodies, we highlight key clinical features to help identify these patients, outline immunological findings that inform laboratory testing and describe the clinically relevant disease biology of relevance to treatment decisions.

### Autoimmune encephalitis is not rare

Until the discovery of neuroglial surface autoantibodies, infections were the most common known causes of encephalitis. However, over the last 20 years, the description of multiple autoantibodies targeting the extracellular domains of neuroglial proteins in patients with encephalitis has shifted this balance. For example, the California Encephalitis Project found that among persons under 30 years of age, N-methyl-D-aspartate receptor (NMDAR)-antibody encephalitis was more common than any individual infectious cause of encephalitis.^5^ Also, autoimmune causes of encephalitis have been reported to be at least as common as viral causes in Olmsted County, USA.^6^ Interestingly, the incidence of autoimmune encephalitis rose in the second 10-year epoch of this study, likely owing to growing awareness of these disorders and more widespread diagnostic capacities. Nevertheless, as fever, focal neurological deficits and cerebrospinal fluid (CSF) lymphocytosis remain inclusion criteria for many ‘all cause encephalitis’ studies, such approaches likely continue to underestimate the prevalence of autoimmune causes, which often lack these features.^7^ In future, we predict that unbiased surveys in patients with encephalitis will show that the growing range of autoimmune causes significantly exceed those of infectious causes in developed countries.

### Distinctive clinical manifestations of individual autoimmune encephalitides

While the clinical features of these disorders span the spectrum of neurological symptomatology, for patients with autoantibodies against any individual target there is often a characteristic set of core phenotypic manifestations, which may relate to the regional expression, function and relative susceptibility of the target protein. Table 1 summarises the most common such syndromes on a ‘per target’ basis.

By way of generalisation, autoantibody-mediated disorders often present rapidly, over a few days to weeks. However, we have observed more chronic courses, of between 1 and 5 years, particularly in leucine-rich glioma-inactivated protein 1 (LGI1)-antibody, contact-associated protein 2 (CASPR2)-antibody and immunoglobulin-like cell-adhesion molecule 5 (IgLON5)-antibody syndromes. These findings mean that time to disease nadir is often outside of the 3-month duration which appears in diagnostic guidelines.^8^ In our clinical experience, these more insidious courses—which are sometimes more akin to neurodegenerative presentations than florid encephalitis syndromes—often lead to a delayed diagnosis, and hence late commencement of immunotherapy. In patients with more acute-onset, dramatic presentations the diagnosis tends to be considered early but immunotherapy may still be delayed while excluding differentials and awaiting autoantibody test results. While tumours, prion disease and metabolic disorders are often in the differential diagnosis, a pragmatic trial of immunotherapy may only be absolutely contraindicated in the setting of some infections. Yet, observational data show that corticosteroids may be beneficial in some forms of herpes simplex virus (HSV) encephalitis, suggesting this may not be a universal contraindication.^9^

**Figure 1:**
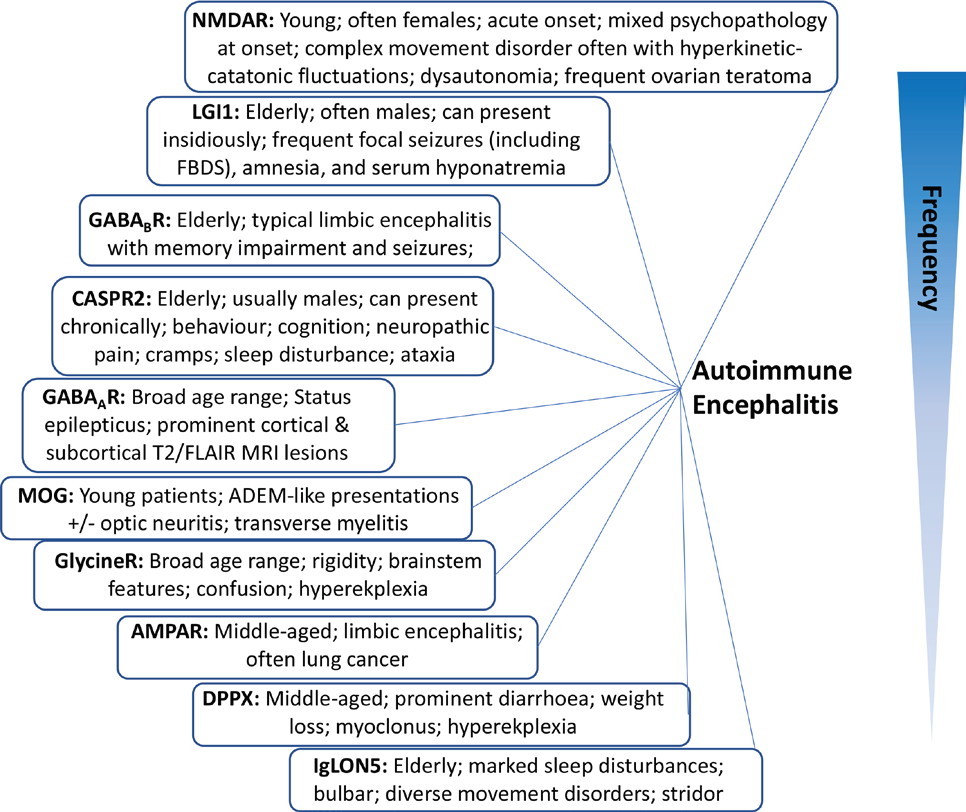
Classic syndromes and characteristic features of neuronal autoantibodies. Listed in an estimated order of descending frequency. AMPAR, α-amino-3-hydroxy-5-methyl-4-isoxazolepropionic acid receptor; CASPR2, contact-associated protein 2; DPPX, dipeptidyl peptidase-like protein 6; GABA_A/B_R, gamma aminobutyric acid; IgLON5, immunoglobulin-like cell-adhesion molecule 5; LGI1, leucine-rich glioma inactivated protein 1; NMDAR, N-methyl-D-aspartate receptor; MOG, myelin-oligodendrocyte glycoprotein.

To encourage earlier immunotherapy administration to these patients, we have set out below some ‘identifying’ clinical findings that we find valuable in everyday autoimmune neurology practice (figure 1). Some features are so characteristic of certain antibody syndromes that they serve as essentially pathognomonic clues to the underlying autoantibody. Later, we describe the dominant presenting features, and relate these to individual syndromes.

#### Psychiatric/behavioural

Psychiatric symptoms such as aggression, irritability, mood lability, hallucinations and marked disturbance in sleep/wake cycles may occur in many of these patients across the spectrum of autoimmune encephalitis, and are especially notable in NMDAR-antibody and α-amino-3-hydroxy-5-methyl-4-isoxazolepropionic acid receptor-antibody syndromes.

In adult-onset NMDAR-antibody encephalitis, psychiatric features are typically the presenting complaint, with patients often needing mental health assessments before a neurology consultation. In our experience, relatively isolated psychiatric features occur in these patients only at disease onset. Subsequently, within a few days, they are rapidly accompanied by more traditional neurological abnormalities including delirium, amnesia and seizures. Nevertheless, careful consideration of the psychopathology can help in differentiating NMDAR-antibody encephalitis from primary psychiatric disease. NMDAR-antibody encephalitis often presents with a complex phenotype spanning classically distinct psychiatric diagnostic categories, including domains of mood, psychosis, behaviour and catatonia, the latter also seen with gamma aminobutyric acid A receptors (GABA_A_R)-antibodies.^10^ By contrast, early ‘transdiagnostic’ presentations are unusual in most primary psychiatric diseases. Overall, the complex psychiatric phenotype at onset combined with polysymptomatic neurological disease and a polymorphic movement disorder, discussed in detail later, creates a multifaceted presentation highly characteristic of NMDAR-antibody encephalitis. These features contrast markedly to the poorly circumscribed clinical syndrome of neuropsychiatric systemic lupus erythematosus, in which NMDAR-antibodies have also been reported. However, by contrast to antibodies which target native neuronal surface epitopes, those from patients with neuropsychiatric systemic lupus erythematosus have been found to show intrinsic ‘stickiness’, which is not NMDAR-specific, and hence have limited diagnostic value.^11^

#### Cognition

In the acute phase, many patients with encephalitis show disorientation, confusion, confabulation and amnesia, features that may relate to the dense expression of many autoantigens in limbic structures, particularly the hippocampus. Patients with LGI1-antibody and NMDAR-antibody syndromes, and other forms of limbic encephalitis, often experience a dense amnesia for the period of acute hospitalisation, especially the nadir of their disease. Some patients and relatives consider this fortuitous due to several, inevitably distressing, events typical of their hospital stays. In LGI1-antibody encephalitis, the amnesia characteristically affects both anterograde memories plus a loss of autobiographical retrograde epochs.^12 13^ Comparative neuropsychological analyses are pending in the other forms of autoimmune encephalitis.

#### Seizures

Seizures occur in most autoimmune encephalitis syndromes and are a common factor that triggers neurological attention. The types and frequencies of seizure vary between autoantibody-mediated diseases and may help pinpoint the individual autoantibody.

In LGI1-antibody encephalitis, the seizure profile is especially well-characterised. These patients, typically men in their fifth to eighth decades, have very frequent focal events with multiple semiologies and only rare generalised seizures. The pathognomonic faciobrachial dystonic seizures are frequent, brief events with posturing of the ipsilateral face and arm that often occur hundreds of times per day.^14 15^ Also, the leg may be involved and the sudden leg spasms often precipitate falls. In addition, patients with LGI1-antibodies may have short-lived, and again frequent, piloerection seizures and experience paroxysmal dizziness spells.^16^ From our experience, paroxysmal dizziness spells are likely ictal events characterised by frequent, intense episodic dizziness without vertigo or electroencephalographic correlates. In these patients, other focal seizure semiologies include more classical temporal lobe events, with rising epigastric phenomenon, sudden onset fear or panic, and déjà-vu or jamais-vu. As many of these are very short lived, they may be subtle and their detection often requires direct questioning of patients and relatives.

Although not as well-characterised as the seizures associated with LGI1-antibodies, CASPR2-antibody encephalitis is also associated with frequent focal seizures and rare generalised seizures.^16^ However, we have not observed faciobrachial dystonic seizures and paroxysmal dizziness spells in the CASPR2-antibody patients, whose seizure semiology awaits further characterisation.

Myelin oligodendrocyte glycoprotein (MOG) antibodies are associated with relapsing syndromes involving brainstem or cortical encephalitis, sometimes with optic neuritis and transverse myelitis, which particularly involve children and young adults. Seizures may present as the index event and the syndrome can evolve to a more diffuse encephalitis, including one which radiologically mimics classical acute disseminated encephalomyelitis. Patients typically respond well to corticosteroid therapies, although the duration of their administration remains controversial as relapses are common.^17–19^ This presentation is rare; in our practice, we have seen one case of MOG-antibody related encephalitis alongside >200 other patients with autoimmune encephalitis.

Status epilepticus may occur in autoimmune encephalitis and is most frequent in patients with antibodies to the GABA_A_R/GABA_B_R. Patients with GABA_A_R-antibody encephalitis frequently have distinctive neuroimaging with cortical and subcortical T2/FLAIR signal on MRI affecting two or more brain regions.^20 21^ In our experience, these multiple ‘fluffy’ lesions appear to be a characteristic feature; their presence consistently associated with GABA_A_R-antibody positivity. Patients with GABA_B_R-antibodies are typically in around their sixth decade of life and commonly present with an acute limbic encephalitis. More rarely, they have a prolonged time course, characterised as a rapidly progressive dementia.^22^ Detection of GABA_B_R-antibodies should prompt a search for malignancy, with tumours in ~50% of patients (most commonly small cell lung cancer).

Although patients with NMDAR-antibody encephalitis often have few seizures, it is sometimes an ictal event that prompts consideration of diagnoses outside the realm of primary psychiatric disease.

One important question is whether testing these autoantibodies benefits a broader population of people with epilepsy. To date, studies have yielded highly divergent positivity rates for autoantibodies in a variety of patients with seizures. However, only recently have studies combined accurate clinical phenotyping with the autoantibody results in unselected populations.^23 24^ These largely concur with our routine clinical experience: patients who have unselected new-onset seizures, neuronal surface autoantibodies and an immunotherapy-responsive syndrome typically have mild features of autoimmune encephalitis, such as cognitive and mood features, specific seizure semiologies, dysautonomia and limbic MRI changes. This clinically-driven assessment approach aims to limit unfruitful or equivocal immunotherapy trials in patients attending epilepsy clinics.

#### Movement disorders

The autoimmune encephalitis syndromes may show a diverse spectrum of movement disorder phenomenologies. In keeping with the complex nature of NMDAR-antibody encephalitis, the associated movement disorder is typically polymorphic, defying classification into classical movement disorder taxonomies.^25 26^ Most characteristically, patients have combinations of chorea, stereotypies and dystonia, with limited tremor, which affect all limbs and—most characteristically—the face and mouth.

Encephalitis syndromes associated with both glycine receptor (GlyR) and dipeptidyl peptidase-like protein 6 (DPPX) antibodies are characterised by hyperekplexia and myoclonus;^27 28^ however, accompanying features, such as marked rigidity and falls in GlyR-antibody encephalitis and prominent diarrhoea in DPPX-antibody encephalitis, can usually differentiate these entities. Although not typically associated with a movement disorder, chorea is rare in LGI1-antibody encephalitis.^29^

Gait disturbances are frequent in CASPR2- and IgLON5-antibody syndromes.^30–32^ IgLON5-antibody disease is associated with a polymorphic sleep disturbance plus progressive supranuclear palsy-like picture with axial rigidity and gait freezing, whereas CASPR2-antibody disease typically has a gait disturbance secondary to episodic or persistent ataxia. Indeed, ataxia helps to differentiate CASPR2- from LGI1-antibody syndromes but, as with psychiatric features and seizures, is rarely the sole clinical manifestation.

#### Dysautonomia

Dysautonomia is a common feature to many of these disorders. These symptoms are typically progressive through the initial disease course and can be life-threatening, requiring close monitoring. Particularly in NMDAR-antibody encephalitis, wide fluctuations in blood pressure and tachy-arrhythmias or brady-arrhythmias are key features that often prompt us to consult with colleagues in intensive care and cardiology. Occasionally, temporary pacing is appropriate. Other autonomic involvement includes orthostatic hypotension, constipation and abnormal sudomotor function.

#### Pain

In our experience, pain is under-recognised in the autoimmune encephalitis syndromes particularly in patients with autoantibodies to CASPR2. In this disease, ~60% of patients report pain.^16 30^ It can occur in the context of a peripheral nerve hyperexcitability syndrome (neuromyotonia, fasciculations, cramps and myokymia) but—more commonly—develops without peripheral motor nerve involvement (Ramanathan, Uy, Bennett and Irani, in revisions). Pain is also less common with LGI1-antibodies.^16 33^ In addition, patients with GlyR-antibodies often complaint of allodynia, dysaesthesia and prominent pruritus.^28^ In all these groups, our experience is that pain may respond partially to immunotherapy but often persists. This area merits more detailed future studies.

## Differential diagnoses

Clinicians need to consider a broad differential diagnosis to reflect the spectrum of neurological phenomenology in autoimmune encephalitis. Here we outline a few considerations that apply in each of several clinical situations.

- Infectious encephalitis (most commonly HSV): often presents with seizures as well as fever, focal neurology and more extensive imaging changes than in autoimmune encephalitis.
- Temporal lobe glioma in cases with mesial temporal swelling: semiologies can overlap but autoimmune encephalitis usually has a less abrupt onset and interval imaging swelling on imaging typically resolves with treatment on interval imaging.
- Creutzfeldt-Jakob disease and other rapid dementias: often remain a differential in more chronic cases, especially patients with LGI1-antibodies. However, in practice, the differences in clinical features, CSF and imaging mean that distinguishing these is usually straightforward.
- Post-ictal MRI changes in patients with frequent seizures can often mimic autoimmune encephalitis in the acute phase.
- Metabolic encephalopathies: usually delirium dominates the clinical picture.
- Hashimoto’s encephalopathy: fundamentally a difficult diagnosis to make as definitions remain unclear. New autoantibody discoveries may better describe many cases once termed ‘Hashimoto’s’.^34^

## Clinical management

### Symptomatic considerations

In addition to treatment of the underlying immunological process, it is often necessary to consider management of seizures, movement disorders, behaviour, pain, sleep and autonomic disturbance, and mood disorders. We do not discuss this substantial topic comprehensively here but rather we focus on special considerations relevant to the two most common forms of autoimmune encephalitis: NMDAR-antibody and LGI1-antibody encephalitis.

The overlap in clinical features between NMDAR-antibody encephalitis and neuroleptic malignant syndrome has led some to hypothesise that patients with NMDAR-antibody encephalitis have hypersensitivity to neuroleptic agents, with an increased risk of developing neuroleptic malignant syndrome.^35–38^ Hence, we judiciously use antipsychotic medications for behavioural symptom management, injury prevention and to facilitate care, often once daily olanzapine 10 mg. Alternatively, we find benzodiazepines are effective, although often at high doses (sometimes up to 180 mg/day of diazepam), for treating both behavioural symptoms and some dyskinesias.^39^ We frequently liaise closely with neuropsychiatry colleagues to manage behavioural features.

As discussed earlier, seizures are a common presenting feature among the autoimmune encephalitis syndromes. However, from 103 patients with LGI1-antibody encephalitis, antiseizure medications alone stopped faciobrachial dystonic seizures in only 10%. By contrast, faciobrachial dystonic seizures stopped within 30 days of starting immunotherapy in 51%, rising to 88% by 90 days.^40^ The same principle appears increasingly true for seizures associated with multiple forms of autoimmune encephalitis.^41^ Thus, it is imperative for appropriate and timely treatment to recognise an underlying autoimmune encephalitis syndrome. Furthermore, patients with LGI1-antibody disease are at higher risk of cutaneous reactions and Stevens-Johnson syndrome with antiseizure medications. Therefore, not only is antiseizure medication use likely to be ineffective but may also result in iatrogenic adverse events. Whenever possible, we prioritise optimisation of immunotherapy in these patients and increasingly reserve antiseizure medications only for generalised convulsions or instances where the seizure semiology is likely to cause injury.

After improvements on immunotherapy, discussed later, patients often ask about the risk of ongoing seizures. Indeed, epilepsy is defined as a tendency to enduring seizures. So, it is of interest that few patients in recent autoimmune encephalitis cohorts developed epilepsy after the acute illness.^41 42^ This observation suggests lifelong antiseizure therapy may not be necessary in many cases. In seizure-free patients keen to stop antiseizure medications, we discuss a trial of weaning including the possible complications of long-term antiseizure medications (eg, osteoporosis, patient choice) and implications for driving.

### Early immunotherapy improves outcomes

The importance of early recognition and diagnosis in autoimmune encephalitis is paramount to the ultimate goal of optimal immunotherapy. Although there are no specific data available for all autoantibody-mediated encephalitis syndromes, the two most common forms of autoimmune encephalitis are clear exemplars where improved patient outcomes associate with early immunotherapy. In LGI1-antibody encephalitis, ~80% of patients noticed that faciobrachial dystonic seizures typically precede onset of marked cognitive impairment. Given that immunotherapy is more effective than antiseizure medications in treating LGI1-antibody-associated seizures, early treatment with immunotherapy has shown great promise for preventing otherwise incipient cognitive impairment and functional disability.^40^ In NMDAR-antibody encephalitis, early treatment independently predicted good outcome (modified Rankin score ≤2) whereas delays in immunotherapy of >4 weeks were associated with poor functional outcomes at 1 year.^43 44^

In NMDAR-antibody encephalitis, teratoma removal is a key step in both acute treatment and relapse prevention.^43^ It is considered of equivalent efficacy to other individual first-line immunotherapies, likely because the teratoma is a germinal centre harbouring NMDAR-reactive B cells.^45^ Men and children tend to have non-paraneoplastic disease. Half of adult female patients are diagnosed with ovarian teratomas. So, especially in these cases, pelvic imaging should be performed, and small or equivocal findings carefully followed up and investigated thoroughly. Repeat serial imaging may be considered in cases where a teratoma is suspected and a clinical relapse should certainly prompt re-investigation. We are familiar with patients in whom the teratoma has been radiologically (mis-)interpreted as a luteal or haemorrhagic cyst. However, overall, most patients do not have a detectable teratoma, meaning that in all cases immunotherapy should not be delayed. Also, in our experiences, empirical oophorectomy is low yield for a microscopic teratoma.

There are several options for acute and long-term immunotherapies in both the inpatient and outpatient settings (table 2). Initial inpatient therapy often involves corticosteroids, intravenous immunoglobulins and/or plasma exchange. While awaiting autoantibody results, we start first-line immunotherapy when we are clinically confident of the diagnosis. Second-line therapies include rituximab, cyclophosphamide and other corticosteroid-sparing agents. Choice of initial therapy should balance the risk profile of the intervention and the severity/trajectory of the individual patient’s disease course.

**Table 2:** Immunotherapeutic options for treatment of autoimmune encephalitis

Immunotherapy	Mechanism of action	Dose/regimen	Monitoring/prophylactic adjunctive therapies	Side-effects
First-line therapy				
Corticosteroids	Multiple: largely via attenuation of immune response via genomic and non-genomic effects.	Methylprednisolone 1 g intravenous daily×3–5 days. +/- Oral prednisone 1 mg/kg/day. Slow steroid taper.	Clinical monitoring for steroids side effects. Calcium, vitamin D±bisphosphonate therapy. Proton pump inhibitor for long steroid tapers. When in combination with other immunotherapy, consider prophylaxis for *Pneumocystis jirovecii*.	Sleep disruption, irritability, osteoporosis, weight gain, hypertension, hyperglycaemia, increased intraocular pressures, upper gastrointestinal bleeding, skin thinning/bruising/ striae, reactivation of chronic infection, suppression of endogenous steroid production,. Rare complications include: avascular necrosis of jaw or hip, *P. jirovecii* pneumonia.
Intravenous immunoglobulin (IVIG)	Very wide to include modulation of T/B-cells, cytokines and innate pathways. Blockade of variable domain of causative antibodies by anti-idiotype antibodies.	2 g/kg intravenous divided over 3–5 days.	Clinical monitoring for allergic reactions, transfusion reactions, aseptic meningitis.	Transfusion reactions (most mild), rare complications include aseptic meningitis, anaphylaxis, acute renal failure, haemolytic anaemia and thromboembolism.
Plasmapheresis	Bulk removal of circulating immunoglobulins. Rebound state may increase susceptibility of circulating antibody-secreting cells and precursors to cytotoxic therapies (ie, cyclophosphamide).	3–5 sessions over 5–10 days.	Clinical monitoring for hypotension, catheter-related complications (thrombosis, infection, air embolism) and anaphylaxis. Monitoring for electrolyte abnormalities throughout.	Mortality 3–5 per 10 000, hypocalcaemia, hypokalaemia, metabolic alkalosis, hypotension, catheter-related complications (thrombosis, infection, air embolism), anaphylaxis, TRALI and rare viral transmission
Second-line therapy				
Mycophenolate	Active metabolite (mycophenolic acid) inhibits inosine-5′-monophosphate dehydrogenase, depletes guanosine nucleotides preferentially in T and B lymphocytes.	Initially 500 mg two times a day, targeting to 1–1.5 g two times a day maintenance.	Before starting: screening for latent HBV, HCV. CBC-D weekly×1 month, q2weeks×2 months→monthly. Electrolytes, Cr/GFR, ALT, AST, ALP, GGT, bilirubin, albumin, INR monthly.	Increased infection risk including reactivation of viral infections (herpes simplex/zoster, polyomavirus (BK virus) associated nephropathy (PVAN), PML and CMV viraemia), increased risk of lymphoma and skin malignancy, cytopenias.
Azathioprine	Inhibition of purine synthesis via active metabolites 6-mercaptopurine and 6-thioguanine.	Initial 50 mg daily, increase by 50 mg increments q1-2 weeks until 2 to 3 mg/kg/day maintenance.	CBC-D weekly×1 month, q2 weeks×2 months→monthly. ALT, AST, GGT, ALP, bilirubin, albumin, INR q3months. Age-related cancer screening and skin checks. Consider testing for thiopurine S-methyltransferase (TMPT) deficiency before initiation.	GI toxicity, dose-related cytopenias, hepatotoxicity, increased infection rates, increased risk of malignancy (including the rare entity hepatosplenic T-cell lymphoma (HSTCL), PML.
Rituximab	Monoclonal antibody against CD20: principally B cell depletion.	Induction: 375 mg/m^2^ intravenous weekly×4 weeks or 500 mg intravenous×2 doses separated by 2 weeks.	Preinitiation: CBC+differential, ALT, AST, LDH, bilirubin, electrolytes, creatinine, screening for latent HBV, HCV, syphilis, HIV, and TB. Monthly postinfusion bloodwork (starting 1-week post-induction): CBC-D, ALT, AST, LDH, bilirubin, electrolytes, Cr.	Mild transfusion-related reactions (headache, fever, chills, nausea), hypotension, anaphylaxis (rare), reactivation of latent infection (TB, hepatitis B).
Cyclophosphamide	Induction of DNA cross-linking and apoptosis by active metabolite (phosphoramide mustard).	750 mg/m^2^ intravenous monthly for 3–6 months.	CBC, HIV, HBV, HCV, VZV, liver enzymes, electrolytes, creatinine+urinalysis weekly for the first 4 weeks, then q 2 weekly for next 2 months→monthly.	Cytopenias (neutropenia most common), nausea/vomiting, diarrhoea, hair loss, mucocutaneous ulceration, haemorrhagic cystitis, infertility, teratogenicity.
Third-line/experimental				
Tocilizumab	Monoclonal antibody against IL-6, blocking binding to IL-6 receptor and preventing IL-6 mediated inflammatory cascade.	Initial: 4 mg/kg intravenous infusion. May increase to 8 mg/kg based on response.	Preinitiation screening for TB. Clinical monitoring for infection. Regular monitoring of blood counts, liver profile and lipids.	Fever response and CRP elevation may be blunted by impairment in IL-6 receptor signalling. Hepatotoxicity, cytopenias, blood lipid abnormalities, immunosuppression.
Bortezimib	Small-molecule proteasome inhibitor. Relatively selective depletion of plasma cells due to high immunoglobulin synthesis rate.			Peripheral neuropathy, myalgia, diarrhoea

Sources: Sun *et al*, Shin *et al*,^2 62 63^ Joint Formulary Committee.

ALP, alkaline phosphatase; ALT, alanine transaminase; AST, aspartate transaminase; CBC-D, complete blood count with differential; CD, cluster of differentiation; Cr, creatinine; CRP, C reactive protein; GFR, glomerular filtration rate; GGT, gamma-glutamyltransferase; HBV/HCV, hepatitis B/C virus; HIV, human immunodeficiency virus; HTLV, human T-lymphotrophic virus; IL, interleukin; TB, tuberculosis; TRALI, transfusion associated lung injury.

In our experience, intravenous corticosteroids are generically highly effective agents, so relative contraindications (eg, pre-existing diabetes or psychiatric diseases) are often carefully managed in the acute phase but rarely considered absolute contraindications. We also find plasma exchange to be very effective, often used if patients show a limited or inadequate response to corticosteroids, or for patients with a rapid deterioration whose trajectory may otherwise be intensive care unit admission. While intravenous immunoglobulin is the only immunotherapy with randomised data to support its use,^46^ in practice it appears the least effective of the three conventional first-line interventions. This observation is supported by the minimal effect size observed in this inaugural randomised control trial.

Below, we discuss our more specific management approaches to the two most common autoantibody-mediated syndromes.

#### NMDAR-antibody encephalitis

Due to its associated high-morbidity and mortality, potential for months of hospitalisation and high rate of relapses, we favour early aggressive therapy in patients with NMDAR-antibody encephalitis. Teratoma removal and first-line immunotherapies are routine interventions: typically, 3–5 days of 1 g intravenous methylprednisolone daily, plus plasma exchange. Second-line immunotherapies reduce the relapse risk and, from our clinical observations, expedite recoveries and time to discharge.^43^ Our threshold to escalate to second-line therapy is increasingly low, with >70% of our patients receiving cyclophosphamide or rituximab if awareness and behaviour have not improved within 2 weeks. As outpatients, we tend not to employ a prolonged course of oral corticosteroids, especially if second-line therapy or tumour removal appears to be having the desired effect. This approach appears to associate with a <5% rate of relapses, to date. If second-line immunotherapy is not administered during initial episode, it should be strongly considered in relapses.

#### LGI1-antibody encephalitis

For this condition, we favour first-line treatment with high-dose intravenous or oral corticosteroids. We have an increasingly low threshold for plasma exchange at disease onset, particularly in patients with greater degrees of impairment. In our experience, oral prednisolone should be maintained for around 24–36 months, as shorter durations of corticosteroids are often associated with relapses.^14^ We typically taper oral prednisolone from 50 to 60 mg for the first 2–4 months to around 20–30 mg by 12 months, with a slow taper thereafter. In elderly patients, this approach does inevitably induce some glucocorticoid side effects that need to be carefully considered. However, in our experience, despite corticosteroid-sparing agents (mainly mycophenolate mofetil) more rapid steroid tapers tend to result in relapse. A few patients who require cyclophosphamide show variable outcomes. By contrast, rituximab appears more effective but longer-term follow-up is awaited.

## Molecular discoveries provide clinical insights

The ability to detect CNS-directed autoantibodies that target the extracellular domains of neuroglial proteins has revolutionised our ability to diagnose and classify this nascent group of autoantibody-mediated disorders. The confident detection of a causative autoantibody has implications for the treatment regimen and may help focus a search for associated malignancies or surveillance for associated complications. Moreover, an understanding of the basic immunobiology helps to appreciate nuances around diagnostic testing, suspected mechanisms of pathogenesis and offer a rationale for administration of therapies. As these diseases are associated with pathogenic autoantibodies, a focus on the B cell immunobiology may be the key to understanding autoimmune encephalitis. A full discussion of the underlying immunopathology is beyond the scope of this review and have been described elsewhere.^2^ Here, we discuss select concepts with the greatest clinical relevance.

### Therapeutic insights

Autoantigen-specific B cells are probably first established peripherally before migrating into the CNS, as the pathogenic neuronal autoantibodies typically have ~50-fold higher concentrations in the serum than in CSF.^2^ Interestingly, this ratio holds true for patients in whom an infectious encephalitis (HSV encephalitis) is followed by an autoimmune form (NMDAR-antibody encephalitis). Therefore, even with a brain-specific trigger, the autoimmunity probably begins outside the CNS. Hence, the peripheral B cells that carry these self-reactivities need to evade tolerance checkpoints, a potential avenue for therapeutic interventions. Also, the B lineage cells that secrete these autoantibodies in the periphery are themselves a key therapeutic target. For example, studies that implicate CD20^−^ long lived plasma cells as dominant producers of autoantibodies imply drugs such as bortezomib—by acting on the proteosome, which is especially active in plasma cells—may be effective treatments.^47^ Alternatively, emerging evidence suggests autoantibodies secreted by CD20^+^ B cells that have undergone recent germinal centre reactions may be a key source of these autoantibodies^45 48^: if this mechanism were dominant, rituximab administration might logically prove to be an especially effective option.

A key factor in generating the mature antigen-specific B cells is their interaction with antigen-specific T cells. This occurs via the engagement of human leucocyte antigen (HLA) with the T-cell receptor. Hence, it remains of biological interest that >90% of patients with LGI1-antibodies carry the HLA-DRB1*07:01 allele, and that ~70% of the patients with CNS diseases and CASPR2-antibodies carry the HLA-DRB1*11:01 allele.^49^ T-cell directed therapies may be a future avenue for treatment in these patients. In addition, these findings may be of value in clinical practice: we have found the absence of these alleles as a useful adjunctive investigation to identify the few patients with LGI1-antibodies or CASPR2-antibodies who do not have an immunotherapy-responsive syndrome. Hence, genetic testing may become a reflexive test in these conditions.

After B cell autoreactivities originate in the periphery, autoantibody access to the CNS is likely to play a major role in pathogenesis. Of course, fundamentally, the autoantibodies must gain access to the brain. But it remains poorly addressed as to whether they cross the blood–brain barrier as soluble immunoglobulins or are predominantly secreted by intrathecal B cells that have crossed the blood–brain barrier. In beginning to address this, recent studies show these patients have an enrichment of autoantigen-reactive B cells in the CSF, providing direct evidence of intrathecal autoantibody production.^50 51^ Hence, drugs that prevent lymphocyte transmigration into the CNS may yet be effective agents in these disorders.

### Diagnostics insights

In addition, the biology around roles of peripheral and central compartments also has implications for diagnostic testing. Autoantibodies can be detected in both CSF and serum, and—put simply*—both* samples should be sent in all patients, wherever possible. However, there are important nuances between conditions. For example, LGI1-antibodies are not detected in around 50% of patient CSF samples.^33^ By contrast, NMDAR-antibodies are consistently detected in the CSF of patients and said to be absent in ~20% of serum samples. Finding autoantibodies in the CSF but not the serum does not seem biologically intuitive given the immunological response likely begins in the periphery, perhaps most clearly in patients with (systemic) ovarian teratomas. By comparison to serum, CSF has a ~500-fold lower total IgG concentration and hence offers a sample with inherently lower backgrounds in diagnostic assays, which may explain the above finding. Yet, in some patients, for example, those who are irritable, not suitable for sedation and in young children, serum may be the only pragmatic sample source. However, serum NMDAR-antibodies occur at ~3% rates in healthy and disease controls and hence so called ‘clinically irrelevant’ serum NMDAR-antibody results are not infrequent, again supporting the use of CSF for detecting NMDAR-antibodies. For these reasons, in this condition, the absence of CSF positivity is considered to indicate a lack of direct autoantibody pathogenicity. However, as described above, the opposite is true for LGI1-antibodies. Therefore, whenever possible, paired CSF-serum should be tested.

**Figure 2:**
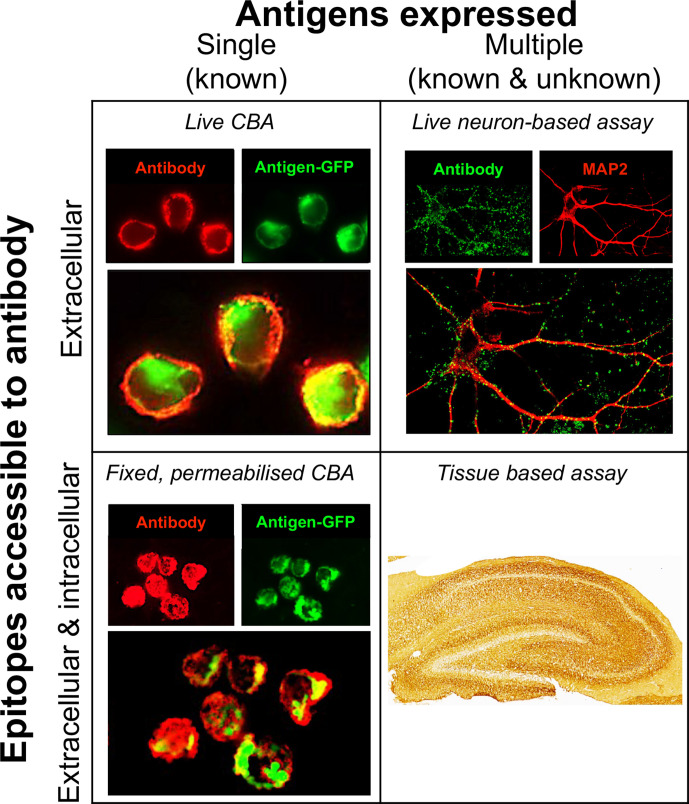
Neuronal surface antibody detection methods. Current research and diagnostic methods expose the test sample to neuronal antigens which differ in the properties of the antigens. Cell-based assays aim largely to expose a single known antigen, by its expression in mammalian cells. Conversely, neurone-based assays and tissue-based assays expose multiple endogenous antigens, both those known to be targets of pathogenic antibodies and as yet unknown antigens. Additionally, the assays vary on whether the antigen was fixed before incubation with the patient sample (serum or cerebrospinal fluid) and whether the cell membrane is intact (‘live’). Live cell-based assays and live neurone-based assays neither fix the surface antigen nor permeabilise the membrane before exposure to the patient’s sample. By contrast, in fixed permeabilised cell-based assays and tissue-based assays, target antigens are potentially altered by fixation and cell membrane integrity is lost. Figure modified from Ramanathan *et al*.^3^ CBA, cell-based assay.

When sending and interpreting results for CNS autoantibody testing, it is important to emphasise the clinical hypothesis. Clinicians interpreting these results should also take into account differences in sensitivity and specificity of individual autoantibody tests (figure 2). For example, several clinical laboratories use commercially available ‘fixed’ cell-based assay kits. These kits have limitations as they inherently alter the native antigens with fixation, creating non-physiological autoantigens.^3^ By contrast, live cell-based assays detect autoantibodies against the closest resemblance of the targets that would be encountered in vivo. Live cell-based assays are often more sensitive than fixed ones^52–54^; therefore, in the setting of an appropriate clinical syndrome, a negative test on fixed cell-based assay should raise suspicion of a false-negative result and clinicians should consider having these samples re-tested at a reference laboratory.

### ‘I’m sure this patient has an autoantibody’

We continue to see several patients with no known autoantibody, but a clinical syndrome compatible with autoimmune encephalitis. In these so called ‘seronegative’ cases, where there is a clinical suspicion of an autoantibody but no identified defined autoantigenic target, we aim to begin early immunotherapy whenever possible given that autoimmune encephalitis is a treatable syndrome. In parallel, we continue to re-evaluate possible alternative diagnoses but escalate therapy when autoimmune encephalitis is considered the likeliest cause.

Various research-level tests can offer greater diagnostic clarity (figure 2).^3^ The patient sera/CSF can be applied to rodent brain sections to identify neuroglial reactivity and, perhaps, a distinctive binding pattern. This approach has been used in several instances as an initial step in target identification, but is also a valuable technique to simply diagnose a brain reactive autoantibody.^55^ As this method exposes patient autoantibodies to both intracellular and extracellular domains of neuroglial proteins, it does not exclusively detect pathogenic species. To define these, it is possible to assess reactivity of serum or CSF IgGs against the surface of cultured neurones or astrocytes. While time consuming to perform, binding patterns have provided valuable information for many patients with suspected autoantibody-mediated syndromes who were negative on available clinical assays. These tests are available on request from research laboratories.

## Closing remarks

The recognition of neuronal surface autoantibodies as a cause of encephalitis has had far-reaching implications. It has helped to define a group of immunotherapy-responsive disorders, describe their pathogenesis, and develop therapies informed by these pathogenic mechanisms. Further, the scope of autoantibody-mediated diseases has expanded beyond the initial limbic encephalitis picture to include other polysymptomatic immunotherapy-responsive syndromes. Clinical suspicion of these disorders remains the cornerstone to their detection and there are now many clinically recognisable syndromes described. Interpretation of autoantibody results should similarly be in the context of this clinical picture. Earlier recognition, treatment and escalation of immunotherapy in many of these syndromes can lead to improved outcomes and reduced disability.

**Further reading**

- Graus F, Titulaer MJ, Balu R, *etal.* A clinical approach to diagnosis of autoimmune encephalitis. *Lancet Neurol* 2016;15:391–404. doi:10.1016/S1474-4422(15)00401-9.A.
- Ramanathan S, Al-Diwani A, Waters P, *etal.* The autoantibody-mediated encephalitides: from clinical observations to molecular pathogenesis. *J Neurol* 2019;1–19. doi:10.1007/s00415-019-09590-9.
- SunB, Ramberger M, O’Connor KC, *etal.* The B cell immunobiology that underlies CNS autoantibody-mediated diseases. *Nat Rev Neurol* 2020;16:481–92. doi:10.1038/s41582-020-0381-z.

**Key points**

- Autoimmune causes of encephalitis are at least as common as infectious causes and should be considered early.
- Several characteristic core phenotypic manifestations may strongly suggest an underlying autoantibody-mediated encephalitis; this should raise the consideration of empiric immunotherapy once infectious causes are reasonably excluded.
- Early immunotherapy improves outcomes in patients with autoimmune encephalitis.
- Whenever possible, paired cerebrospinal fluid and serum should be tested, and clinicians should emphasise the clinical hypothesis when interpreting the results.
- Brain sections and neuronal cultures are valuable methods to detect autoantibodies in patients who have a suspected autoimmune condition despite negative antigen-specific results.
